# Prognostic Impact of Lymphoid Enhancer Factor 1 Expression and Serum Galectin.3 in Egyptian AML Patients

**DOI:** 10.1155/2019/2352919

**Published:** 2019-12-18

**Authors:** M. A. ElBaiomy, S. Aref, M. El Zaafarany, Sara Atwa, Tamer Akl, Wafaa El-Beshbishi, Shaimaa El-Ashwah, L. Ibrahim, M. El-Ghonemy

**Affiliations:** ^1^Medical Oncology Unit, Mansoura University Oncology Center, Mansoura, Egypt; ^2^Hematology Unit, Clinical Pathology Department, Mansoura University, Egypt; ^3^Clinical Oncology and Nuclear Medicine Department, Mansoura University, Egypt; ^4^Clinical Hematology Unit, Mansoura University Oncology Center, Mansoura, Egypt

## Abstract

**Background:**

Deregulation of the Wnt signaling pathway had a role in haematological malignancies. Previous studies reported that lymphoid enhancer factor 1 (LEF1) expression and serum Galectin-3 level could affect clinical parameters and outcome in acute myeloid leukemia patients, but as far as we know, no study has addressed their combined effect on AML patients.

**Aim:**

We studied the expression of LEF1 by real-time qPCR and measured serum level of Gal.3 by ELISA technique in peripheral blood of 69 AML patients and correlated it with different clinicopathological criteria of patients, response, PFS and OS.

**Results:**

We found high expression (LEF1^high^) was associated with better OS (*p* = 0.02) and EFS (*p* = 0.019) compared to LEF1^low^, low serum Gal.3 level had better OS (*p* = 0.014) and EFS (*p* = 0.02) compared to high serum Gal.3 level. LEF1^high^ less likely to carry a FLT3-ITD (*p* = 0.047) compared to LEF1^low^ patient, also LEF1^high^ characterized by favorable risk (*p* = 0.02) than LEF1^low^ patients. While patients with higher Gal-3 levels characterized by poor risk (*p* = 0.02) than lower Gal.3 lels, also more likely to carry a FLT3-ITD with borderline significance (*p* = 0.054). Combined LEF1^high^/Gal.3 low patients had lower baseline blast percentages (*p* = 0.02), favorable risk (*p* = 0.01), less likely to carry FLT3-ITD (*p* = 0.02), higher CR rate (*p* = 0.055), shorter time to CR (0.001) than other groups. Among high Gal.3 level group, LEF1^high^expression improved OS and EFS (20 and 15 months respectively) vs LEF1^low^ expression (13 and 8 months respectively).

**Conclusion:**

We conclude that high LEF1 expression was a favorable prognostic marker which can define AML patient risk and outcome independent from assessing the serum galectin.3 level.

## 1. Introduction

The Wnt signaling is critically involved in normal haematopoietic development and self-renewal process of haematopoieic stem cells (HSCs) [1]. The Wnt signaling pathway is frequently dysregulated leading to more cancer susceptibility [2].

The function of *β* catenin is under control of Wnt pathway, in case of absence of Wnt ligand, *β* catenin will be primed for degradation by proteasomes through its phosphorylation by destruction complex (GSK3*β*, CK1, Axin and APC), on the other hand when Wnt ligand binds its receptor (Frizzled and LRP5/6) the level of indestructible phosphorylated *β* catenin will increase saturation of the dendritic cells (DC) leading to cytosolic accumulation of nonphosphorylated *β*-catenin which translocate to the nucleus forming complexes with the *T*-cell factor (TCF)/lymphoid enhancer factor (LEF) transcriptional regulators and also promotes activation of proto-oncogenic Wnt target genes like c-myc, cyclinD1 and tumor survival [3].

Galectin-3 (gal-3) is a glycoprotein that has a role in fibrosis, inflammation, and cancer [4]. Gal-3 overexpression upregulates cyclin D1 and c-myc, and is also a novel binding partner of *β*-catenin and is phosphorylated, like *β*-catenin, by casein kinase I [5]. Studies showed that elevated gal-3 level is a poor prognostic factor in different solid tumors as prostate cancer [6], melanoma [7] and haematological disorders as nonhodgkin lymphoma (NHL) [8], multiple myeloma [9] and acute myeloid leukemia patients [10].

LEF1 expression dysregulation have a more complex role independent of Wnt signaling result in several disease patterns. Up regulation of LEF1 had a poor outcome in *T*-cell lymphomas [11], while in adult AML associated with favorable outcomes [12].

In the present study, we focused on Gal.3 and LEF1, which have been described as key mediators of Wnt pathway in which dysregulation of this pathway has been associated with AML pathogenesis and prognosis.

## 2. Patients and Methods

### 2.1. Patients

This study was carried on 69 AML patients recruited at oncology Mansoura university center and Metghamr Oncology Center, in addition to twenty healthy subjects matched in age and sex as reference control. Diagnosis of AML established according to 2008 WHO diagnostic criteria [13]. The study was approved by the institutional review board and all patients provided written informed consent. Follow up was up to three years to assess prognosis and outcome.

AML cases were treated by cytarabine based intensive chemotherapy regimen with different dosages during induction therapy based on performance status. Relapsed/ refractory cases were treated by either HAM (high dose cytarabine and mitoxantrone) or FLAG (fludarabine, cytarabine and G-CSF) protocol.

### 2.2. Methods

#### 2.2.1. Measurement of Galectin-3 Level

Serum samples were taken from the patients and the control group. Serum level of galectin 3 was measured using ELISA technique.

#### 2.2.2. Measurement of LEF1 Expression by Quantitative PCR

The LEF1 was amplified by real-time qPCR from cDNA after reverse transcription of mRNA. LEF1 expression was measured using a Taqman probe-based qPCR assay recognizing all four major human LEF1 isoforms (Hs01547250_m1; Applied Biosystems, Foster City, CA), and normalized to GAPDH gene expression to allow comparison of our expression data [14].

## 3. Statistical Analysis

The statistical analysis was done using Excel 2007 program and SPSS version 16 (Statistical Package for Social Science). Qualitative data were described in the form of numbers and percentages. Quantitative data were described in the form of mean (±) standard deviation (SD). Statistical analysis was done by comparison between groups using chi-squared test regarding qualitative data, while quantitative nonparametric data comparison was performed using one-way ANOVA and paired sample *t*-test. Survival analysis was calculated by the Kaplan-Meier product-limit estimator. Comparison of the survival was performed by the log-rank test; continuous variables were dichotomized at the median cutoff. The probability of being by chance (*p* value) was calculated for all parameters (*p* is significant if <0.05 or =0.05 at confidence interval 95%).

## 4. Results

Sixty nine patients with AML (36 M; 33 F) were included in our study, their mean age was 45.74 years ± 15.98 (ranging from 18 to 81 years), Descriptive data of studied patients were illustrated in Table 1. LEF1 expression and serum galectin.3 in AML patients were significantly higher than control.

### 4.1. Association of LEF1 and Galectin-3 Expression Levels with Baseline Patients Characteristics

AML patients with LEF1^high^ expression had lower pretreatment white blood cell counts (*p* = 0.047), higher platelet level reaching significance (*p* = 0.057), lower BM blasts percentages (*p* = 0.012) compared to LEF1^low^ patients. LEF1^high^ less likely to carry a FLT3-ITD (*p* = 0.047) compared to LEF1^low^ patients, also LEF1^high^ characterized by favorable risk (*p* = 0.02) than LEF1^low^ patients. LEF1 expression did not differ significantly with respect to age, haemoglobin level and NPM mutations (*p* > 0.05) (Table 2). While patients with higher Gal-3 levels characterized by poor risk (*p* = 0.02) than lower Gal.3 levels, also more likely to carry a FLT3-ITD with borderline significance (*p* = 0.054), meanwhile Gal.3 levels did not differ significantly regarding age, WBC, haemoglobin level, platelets, blasts percentage and NPM mutations (*p* > 0.05) (Table 2).

### 4.2. The Association between LEF1, Galectin-3 Level and Treatment Outcome

LEF1^high^ patients achieved a significantly higher CR rate (61.4% vs. 38.6%, *p* = 0.028) with a shorter time to CR (34.84 ± 15.7 vs. 43.5 ± 13.2, *p* = 0.036) than LEF1^low^, while refractory/relapsed cases were not affected by high vs low LEF1 expression (*p* = 0.3). High Gal.3 level were associated with significantly lower first CR (42.1% vs. 57.9%, *p* = 0.01) and a longer time to CR (50.21 ± 11.91 vs. 29.42, *p* = 0.001) than lower Gal.3 levels, while Gal.3 level showed no difference regarding refractory/ relapsed cases (*p* = 0.6) (Table 3).

### 4.3. Influence of Low Gal.3/LEF1^high^ vs. Others on Baseline Patients' Characteristics and Treatment Outcome

We found that in a subset group of patients with combined LEF1^high^ and low Galectin.3, they could have a different disease behavior and we found that LEF1^high^/Gal.3 low patients with lower blast percentages (*p* = 0.02) were less likely to be presented with extramedullary disease at diagnosis (*p* = 0.065), favorable risk (*p* = 0.01), less likely to carry FLT3-ITD (*p* = 0.02) and with more rebound thrombocytosis after induction chemotherapy (*p* = 0.01) versus other groups, Table 4.

### 4.4. LEF1 Expression and Gal.3 Level and Survival Outcome

**Figure 1:**
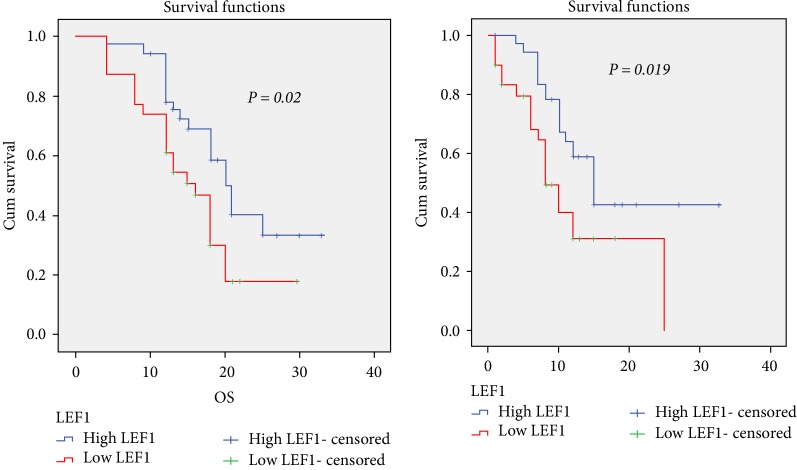
High LEF1 expression was associated with better EFS & OS in AML patients.

**Figure 2:**
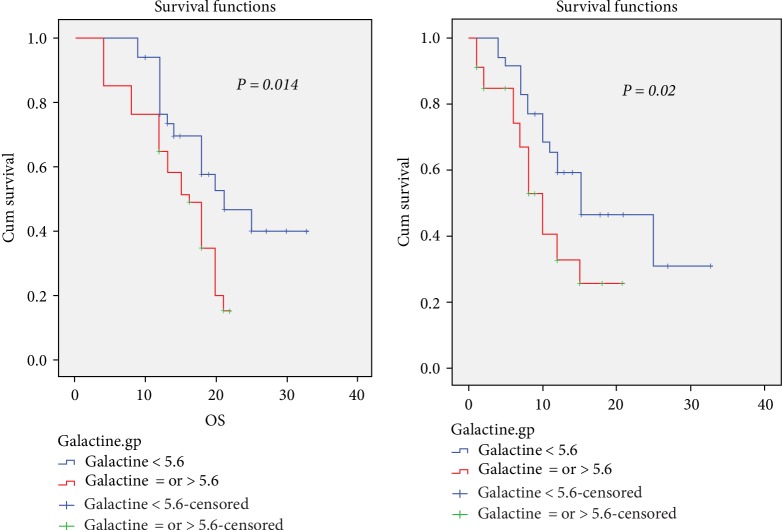
Low Gal.3 level was associated with better EFS & OS in AML patients.

**Figure 3:**
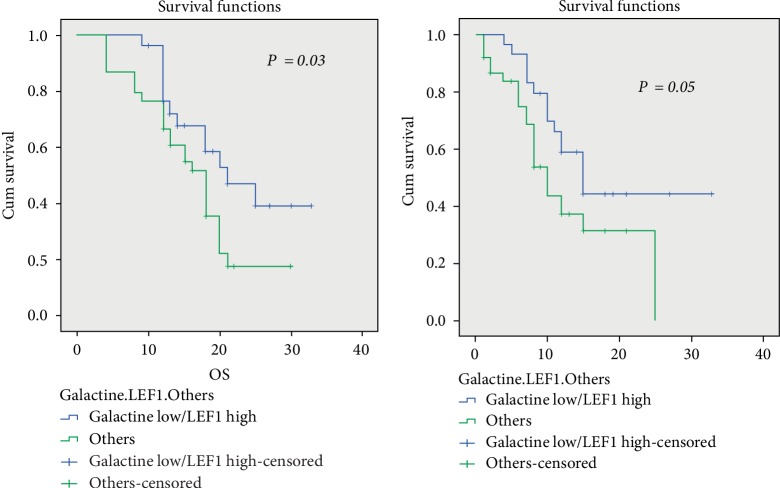
HFL1^high^/low Gal.3 level were associated with better EFS & OS in AML patients.

Kaplan–Meier survival analysis revealed that LEF1^high^ patients had a significantly longer overall survival (OS) (*p* = 0.02, Figure 1) and better event free survival (EFS) (*p* = 0.019, Figure 1) than LEF1^low^ patients. High Gal.3 had a significantly shorter OS (*p* = 0.014, Figure 2) and EFS (*p* = 0.02, Figure 2) than low Gal.3 level. LEF1^high^/low Gal.3 level patients had a significantly longer OS (*p* = 0.03, Figure 3) and borderline significantly better EFS (*p* = 0.05, Figure 3) than others (Table 5).

## 5. Discussion

AML is the most common acute leukemia in adults, about 80% of cases in this group [15]. Although clinical factors (age and performance status) have an important role in treatment guide, cytogenetic changes considered the strongest predictor for outcome and also gene mutations have helped further refine risk stratification especially in cytogenetic normal AML [16].

Despite the advances in AML therapy that have led to significant improvements in outcomes for younger patients, poor prognosis remains a major concern in the elderly whom account for the majority of new cases [17].

To our knowledge, our data is the first study to evaluate the impact of LEF1 expression within the two Gal.3 level groups separately and demonstrated that LEF1^high^ and low Gal.3 level patients were associated with favorable risk, better outcome in AML compared to others.

Previous studies suggest that down-regulated LEF1 in adult B-precursor acute lymphoblastic leukemia [18] and chronic lymphocytic leukemia (CLL) associated with favorable outcome [19]. However, other studies showed that low LEF1 level is a poor prognostic factor in myelodysplastic syndromes, AML [20], acute promyelocytic leukemia of adult patients [21], and childhood ALL [22].

In present study we found that LEF1^low^ expression AML patients had higher WBC, BM blast percentages, lower platelets level, also had poor cytogenetic, and carried FLT-3 mutation. In agreement with our results, Metzeler et al., [14] conducted a study on 210 cytogenetically normal (CN)—AML patients, also by Albano et al. [21]. Fu et al. (2014) showed no significant difference regarding age, WBC, FLT3-ITD mutations between LEF1 expression in 101 AML patients.

We further demonstrated that significant number of patients in LEF1^high^ group achieved CR rate which was influenced by baseline WBC, BM blast percentage, cytogenetic risk and FLT3 mutation status, also associated with significantly shorter time to CR which reduce the duration of hospital admission, post-induction therapy compared to LEF1^low^ patients. Similarly the Chinese study conducted by Fu et al. (2014) showed CR rate was higher in LEF1^high^ patients than LEF1^low^ patients and also LEF1 level were markedly decreased after induction [12, 24].

We demonstrated that LEF1^low^ expression AML patients had significantly shorter OS and EFS, similar to Albano et al. and Metzeler et al., while Fu group showed that no differences in OS and RFS between 2 groups.

While, Salarpour et al. rebutted this finding and showed that LEF1 gene was down regulated significantly in 96 Iranians AML patients and LEF1^low^ expression was associated with increased WBC and blast percentage through differentiation arrest in AML blast cells. This difference could be attributed to different ethnic and genetic background of studied cases.

The cause behind abnormal LEF1 expression and its effect is more likely based on the cellular context and differentiation stage, putting in mind reports linked Wnt pathway activation and increased *β*-catenin levels with inferior patient outcomes [25], it might sound more reasonable that increased levels of the *β*-catenin interaction partner LEF1 associate with favorable outcomes in AML.

However, Simon et al. [26] showed that, no relation between LEF1 expression and Wnt pathway activation, and [14] demonstrated that FLT3-ITD mutations associated with low LEF1 expression, meanwhile the Wnt pathway is dysregulated in such patients. Also, LEF1 is implicated in several other cellular pathways as CEBPA down-regulation and neutrophilic differentiation block. Thus Low LEF1 expression may involve arrest of blast cells differentiation in MDS and AML, as reflected by the higher WBC and blast percentages in LEF1 low CN-AML and MDS [27]. And finally, LEF1 expression has a role in granulopoiesis and lymphocyte development [28].

Various reports had found a role of Gal.3 expression in pathogenesis and progression of solid tumor as prostate and bladder cancer [29, 30]. Meanwhile the relationship between galectin-3 expression and outcome in haematological malignancies remain controversial [31]. Gal.3 has anti-apoptotic effect when localized in cytosol, while in the nucleus it has a pro-apoptotic effect [32]. Asgarian-Omran et al. [33] demonstrated that galectin 3-mRNA was dramatically reduced allowing mature lymphocytes to escape from apoptosis in the advanced stage CLL patients. In contrast, increased serum galectin-3 expression may be associated with worse prognosis in primary central nervous system lymphoma [34] NHL [35] and AML [36]. Meanwhile, Yamamoto-Sugitani et al. [31] had reported that gal-3 is predominantly expressed in CML cells, but not in acute leukemias.

Our data revealed that low Gal.3 level had favorable risk cytogenetic, OS and EFS than high Gal.3 patients similar to data reported by Cheng et al. [36] and Gao et al. [10]. Also our patients with high gal.3 were associated with significantly lower CR rate compared to low Gal.3 level, which was explained by Hu et al. [37], that high gal-3 level resulting in chemotherapy resistance in acute leukemia in vitro cell lines and vivo studies via activating target genes, cyclin D1, c-Myc and surviving.

In our study, the median follow up duration of studied cases was 15 months range (4–33 months). LEF1^high^ was associated with better OS (*p* = 0.02) and EFS (*p* = 0.019) compared to LEF1^low^, low Gal.3 level had better OS (*p* = 0.014) and EFS (*p* = 0.02) compared to high Gal.3 level. LEF1 expression differed between the two Gal.3 groups; they were 43.5% LEF1^high^/low Gal.3, 37.7% LEF1^low^/high Gal.3, 11.6% LEF1^high^/high Gal.3, 7.2% LEF1^low^/low Gal.3. Among high Gal.3 level group, LEF1^high^ expression improved OS and EFS vs. LEF1^low^ expression.

We conclude that high LEF1 expression is a favorable prognostic marker which can define AML patient risk and also outcome independent from assessing the serum galectin.3 level.

## Figures and Tables

**Table 1:** Baseline patients characteristic.

Characteristic	Value
Age (Mean ± SD, range)	45.74 + 15.98 (18–81)

Male, *n* (%)	36 (52.2%)

WBC × 10^9^/L (Mean ± SD, range)	73.19 ± 72.95 (0.6–300)

HB g/L (Mean ± SD, range)	8.01 ± 1.95 (3.8–14.2)

Plt × 10^9^/L (Mean ± SD, range)	54.27 ± 50.18 (3–320)

BM blast% (Mean ± SD, range)	69.52 ± 22.58 (25–100)

*FAB subtypes, n (%)*	
M0	1 (1.4%)
M1	7 (10.1%)
M2	27 (39.1%)
M4	13 (18.8%)
M5	19 (27.5%)
M6	2 (2.9%)

*FLT3.ITD mutation status, n (%)*	
Unmutated	40 (58%)
Mutated	19 (27.5%)
Missing data	10 (14.5%)

*NPM1 mutation status, n (%)*	
Unmutated	39 (56.5%)
Mutated	20 (29%)
Missing data	10 (14.5%)

*Risk, n (%)*	
Favorable risk	17 (24.6%)
Intermediate risk	23 (33.3%)
Poor risk	19 (27.5%)
Missing data	10 (14.5%)

*Response n (%)*	
Achieve 1st complete response (CR)	57 (82.6%)

*Relapse/refractory disease, n (%)*	
Relapse/refractory	38 (55.1%)

**Table 2:** Baseline characteristics in AML patients according to LEF1 and Gal-3 expression levels.

	LEF1			*P*	Galectine.3			*P*
	Low, *n* = 31	High, *n* = 38			<5.6, *n* = 35	≥ 5.6, *n* = 34		
Age	46.68 ± 16.65	44.97± 15.59	−0.4	0.6	46.49 ± 16.06	44.97 ± 16.09	0.3	0.6
WBC × 10^9^/L	92.43 ± 82.07	57.51± 9.94	−2.02	0.047	71.61 ± 73.68	74.83 ± 73.26	−0.1	0.6
Hb gm/dl	8 ± 1.74	8.02 ± 2.13	0.03	0.9	7.99 ± 2.13	8.03 ± 1.78	−0.8	0.9
Plt × 10^9^/L	43.91 ± 33.46	66.96 ± 63.43	−1.9	0.057	63.9 ± 62.5	44.88 ± 32.44	−1.5	0.1
BM blast %	77 ± 19.17	63.42 ± 23.52	−2.5	0.012	64.54 ± 23.19	74.65 ± 21.04	−1.8	0.06
FLT3-ITD mutated	12 (63.2%)	7 (36.8%)	4.9	0.047	6 (31.6%)	13 (68.4%)	4.1	0.054
NPM1 mutated	7 (35%)	13 (65%)	0.6	0.5	11 (55%)	9 (45%)	0.2	0.7
Favorable risk	3 (17.6%)	14 (82.4%)	7.6	0.02	13 (76.5%)	4 (23.5%)	7.3	0.02
Intermediate risk	10 (43.5%)	13 (56.5%)			11 (47.8%	12 (52.2%)		
Poor risk	12 (63.2%)	7 (36.8%)			6 (31.6%)	13 (68.4%)		

**Table 3:** The association between LEF1, Galectin-3 and treatment outcome.

	LEF1			*P*	Galectine.3			*P*
	Low, *n* = 31	High, *n* = 38			<5.6, *n* = 35	≥5.6, *n* = 34		
Time to 1^st^ CR	43.5 ± 13.23	34.84 ± 15.71	−2.1	0.036	29.42 ± 10.92	50.21 ± 11.91	−6.8	0.001
Complete response	22 (38.6%)	35 (61.4%)	5.3	0.028	33 (57.9%)	24 (42.1%)	6.7	0.01
Relapse/refractory	19 (50%)	19 (50%)	0.8	0.3	18 (47.4%)	20 (52.6%)	0.3	0.6

**Table 4:** Prognostic impact of LEF1 expression among two Gal.3 level groups.

	LEF1^high^/Galectine.3 low (30)	Others (39)		*P*
Age	46.43 ± 16.49	45.21 ± 15.77	0.3	0.6
Performance status (PS 2&3)	7 (41.2%)	10 (58.8%)	0.04	0.5
WBC × 10^9^/L	59.94 ± 66.87	83.39 ± 76.59	−1.3	0.1
Hb gm/dl	7.88 ± 2.04	8.1 ± 1.9	−0.4	0.6
Plt × 10^9^/L	44.53 ± 34.24	61.76 ± 58.98	1.4	0.1
BM blast %	62.37 ± 23.77	75.03 ± 20.23	−2.3	0.02
Extramedullary disease	5 (26.3%)	14 (73.7%)	3.1	0.065
FLT3-ITD mutated	4 (21.1%)	15 (78.9%)	6.02	0.02
NPM1 mutated	9 (45%)	11 (55%)	0.01	0.9
Favorable risk	12 (70.6%)	5 (29.4%)	8.9	0.01
Intermediate risk	10 (43.5%	13 (65.5%)		
Poor risk	4 (21.1%)	15 (78.9%)		
Time to 1st CR	29.50 ± 11.81	46.55 ± 2.53	−5.04	0.001
Complete response	28 (49.1%)	29 (50.9%)	4.2	0.055
Rebound thrombocytosis after induction	20 (58.8%)	14 (41.2%)	6.4	0.01
Relapse/refractory	15 (39.5%)	23 (60.5%)	0.5	0.4

**Table 5:** Univarate analysis of risk factors for overall survival and event free survival in AML.

	Overall survival				Event free survival			
	Median (months)	95% CI	Log rank	*P* value	Median (months)	95% CI	Log rank	*P* value
LEF1 low (31)	16	12.5–19.4	5.4	0.02	8	5.6–10.3	5.5	0.019
LEF1 high (38)	20	16.9–23.01			15	11.6–18.3		
Galectine.3 <5.6 (35)	21	12.9–29.01	6.07	0.014	15	6.6–23.3	4.7	0.02
Galectine.3 ≥5.6 (34)	16	12.3–19.6			10	7.8–12.1		
Galectine.3 low/LEF1 high (30)	21	13.4–28.5	8.2	0.04	15	10.9–19	7.7	0.052
Galectine.3 low/LEF1 low (5)	18	4.9–31.5			12	4.1–27.3		
Galectine.3 high/LEF1 high (8)	20	15.9–24.02			15	4.7–25.2		
Galectine.3 high/LEF1 low(26)	13	8.2–17.7			8	5.5–10.4		

## Data Availability

The data used to support the findings of this study are available from the corresponding author upon request.
